# Hemoglobin A1c Threshold for Reduction in Bone Turnover in Men With Type 2 Diabetes Mellitus

**DOI:** 10.3389/fendo.2021.788107

**Published:** 2021-12-28

**Authors:** Sabaa Joad, Elliot Ballato, FNU Deepika, Giulia Gregori, Alcibiades Leonardo Fleires-Gutierrez, Georgia Colleluori, Lina Aguirre, Rui Chen, Vittoria Russo, Virginia Carolina Fuenmayor Lopez, Clifford Qualls, Dennis T. Villareal, Reina Armamento-Villareal

**Affiliations:** ^1^ Division of Endocrinology Diabetes and Metabolism, Baylor College of Medicine, Houston, TX, United States; ^2^ Department of Medicine, Michael E. DeBakey VA Medical Center, Houston, TX, United States; ^3^ Division of Endocrinology, University of New Mexico, Albuquerque, NM, United States; ^4^ Research Service Line, New Mexico VA Health Care System, Albuquerque, NM, United States; ^5^ Biomedical Research Institute of New Mexico, Albuquerque, NM, United States

**Keywords:** Hemoglobin A1c, type 2 diabetes mellitus, bone turnover markers, CTX, osteocalcin

## Abstract

**Background:**

Emerging data suggest that type 2 diabetes mellitus (T2D) is associated with an increased risk for fractures despite relatively normal or increased bone mineral density (BMD). Although the mechanism for bone fragility in T2D patients is multifactorial, whether glycemic control is important in generating this impairment in bone metabolism remains unclear. The purpose of our study is to identify a hemoglobin A1c (A1c) threshold level by which reduction in bone turnover begins in men with T2D.

**Method:**

A cross-sectional analysis of baseline data was obtained from 217 men, ages 35–65, regardless of the presence or absence of hypogonadism or T2D, who participated in 2 clinical trials. The following data were obtained: A1c by HPLC, testosterone and estradiol by LC/MS, bone turnover markers Osteocalcin [OC], C-terminal telopeptide [CTx], and sclerostin by ELISA, and BMD by DXA. Patients were grouped into 4 categories based of A1c (group I: <6%, group II: 6.0–6.4%, group III: 6.5–6.9%, and group IV: ≥7%). Threshold models were fit to the data using nonlinear regression and group comparisons among the different A1c categories performed by ANOVA.

**Results:**

Threshold model and nonlinear regression showed an A1c cut-off of 7.0, among all choices of A1cs, yields the least sum of squared errors. A comparison of bone turnover markers revealed relatively lower OC (p = 0.002) and CTx (p = 0.0002) in group IV (A1c ≥7%), compared to the other groups. An analysis of men with T2D (n = 94) showed relatively lower OC (p=0.001) and CTx (p=0.002) in those with A1c ≥7% compared to those with <7%, respectively. The significance between groups persisted even after adjusting for medications and duration of diabetes.

**Conclusion:**

An analysis across our entire study population showed a breakpoint A1c level of 7% or greater is associated with lower bone turnover. Also in men with T2D, an A1c ≥7% is associated with low bone turnover.

## Introduction

It is well-recognized that patients with type 2 diabetes mellitus (T2D) are at an increased risk for fractures despite normal or relatively higher BMD (1–3). Although there are multiple mechanisms hypothesized as contributors to bone fragility in patients with T2D such as poor glucose control, accumulation of advanced glycation end products (AGEs), use of antidiabetic medications, and the presence of microvascular complications (2, 4), it is well-established that these individuals have suppressed bone turnover, primarily reduced bone formation (5–7). At this juncture, it remains unclear how glycemic control by itself influences bone turnover in the presence or absence of antidiabetic medications (6, 8). *In vitro* studies have shown that hyperglycemia is toxic to the osteoblast leading to reduced bone formation (8), which ultimately also results in reduced bone resorption. It is recommended by the ADA to maintain an A1c of less than 7% to prevent microvascular and other complications from diabetes; however, there is no data that identifies an A1c threshold level for impairment in bone metabolism in patients with T2D. The bone is a dynamic organ and changes in bone turnover precede alterations in bone structural and biomechanical properties. The objective of this study is to identify the hemoglobin A1c (A1c) threshold by which reduction in bone turnover begins in the entire population of male volunteers and subsequently analyze the effect of this threshold on bone turnover in men with T2D. We hypothesize that the reduction in bone turnover is influenced by glycemic control; those with worse control will have greater suppression in bone turnover, while well-controlled diabetics will have bone turnover markers similar to non-diabetics. Furthermore, we hypothesize that there is a cut-off of A1c level at which this abnormality in bone turnover commences and above which bone turnover markers will show significant reduction.

## Methods

### Study Population

This is a secondary analysis using the baseline data from 2 clinical trials in veterans who volunteered to be screened for the study evaluating the effect polymorphisms in the CYP19A1 gene on the response to testosterone therapy in men with hypogonadism (NCT: 01378299) between October 2011 to November 6, 2016 (9, 10) and part of the population of men who screened between May 2018 to October 2019 for the study on the effect of aromatase inhibitors and weight loss in obese men with hypogonadism (NCT: 03490513) (11, 12). There were 105 men from the former and 112 from the latter study were included in this analysis. Inclusion criteria in both studies have been published elsewhere, but briefly, the first one included men between 40 and 75 y who have an average fasting total testosterone (T) level from 2 measurements taken between 8 and 11 AM on 2 occasions 30 min apart of less than 300 ng/dl and with no medical problems that may prevent them from finishing the study. Exclusion criteria included: treatment with bone-acting drugs (e.g., bisphosphonates, denosumab, teriparatide, glucocorticoids, sex-steroid compounds, selective estrogen receptor modulators, androgen deprivation therapy, and anticonvulsants) and finasteride. Additional exclusion criteria included: osteoporosis and history of fragility fractures or diseases known to affect bone metabolism such as: hyperparathyroidism, chronic liver disease, uncontrolled or untreated hyperthyroidism, and significant renal impairment (creatinine of >1.5 mg/dl). Those with a history of prostate cancer, breast cancer, and untreated sleep apnea also met the criteria for exclusion. The inclusion criteria for the second study include men between 35 and 65 y with BMI of 35 kg/m^2^ or more who have an average fasting total T level from 2 measurements of less than 300 ng/dl, taken between 8 and 10 AM on 2 separate days within 1 month and with symptoms consistent with androgen deficiency as assessed by the quantitative Androgen Deficiency in Aging Male (qADAM) questionnaire. LH should be less than 9.0 mIU/L and estradiol (E2) should be 14 pg/ml or more. Since the primary study includes lifestyle intervention to promote weight loss by dietary behavioral modification and supervised exercise program in addition to either aromatase inhibitors or placebo, those with: 1) cardiopulmonary disease (e.g., recent myocardial infarction or MI defined as MI within 6 months at the time of study entry, unstable angina, and stroke) or unstable disease (e.g., NYHA Class III or IV congestive heart failure), severe pulmonary disease requiring steroid pills or the use of supplemental oxygen (that would contraindicate exercise or dietary restriction), unstable weight (i.e., ± 2 kg) in the last 3 months, and diabetes mellitus with a fasting blood glucose of more than 160 mg/dl, and/or Hemoglobin A1c (A1c) more than 9.5% were excluded from participation and clinical/biochemical evidence of pituitary or hypothalamic disease. The rest of exclusion criteria were as the first study as detailed above. Based on the review of baseline data of participants from the above two studies, our study included a mix of hypogonadal (n = 134) and non-hypogonadal men (n = 83).

All participants provided written informed consent in accordance with the guidelines in the Declaration of Helsinki for the ethical treatment of human subjects. The study was conducted at the New Mexico VA Health Care System and at the Michael E. DeBakey VA Medical Center. The protocol was approved by the Institutional Review Boards of the University of New Mexico and of the Baylor College of Medicine. Participants were recruited from patients attending the Endocrine, Urology and Primary Care Clinics of the New Mexico VA Health Care System and the Michael E. DeBakey VA Medical Center. Recruitment was accomplished either through flyers or letters to physicians about patients who may qualify for the study.

### Bone Mineral Density (aBMD) by Dual Energy X-Ray Absorptiometry (DXA)

BMD was measured by DXA of lumbar spine and proximal femur using Hologic Discovery (Hologic Inc, Bedford, MA, USA). Regions of interest in the lumbar spine include L1–L4 vertebrae while that of the femur include the total hip and femoral neck. The coefficients of variation (CV) at our center are ~1.1% for the lumbar spine and 1.2% for the proximal femur (13).

### Type 2 Diabetes Mellitus

The presence of T2D was ascertained from diagnosis in the chart, the intake of medication for T2D, Hemoglobin A1c values ≥6.5% and fasting blood glucose of ≥126 mg/dl. Definition of T2D was made if at least of one these rules is present (6, 10).

### Biochemical Measurements

Fasting blood samples were collected at baseline; serum samples were extracted and stored at −80°C until analysis. Baseline serum T levels represent an average of 2 determinations measured at the end of the study, by liquid chromatography/mass spectrometry (LC/MS) (Mayo Clinic Laboratories, Mayo Clinic, Rochester, MN). Testosterone intra-assay CVs are 7.4, 6.1, 9.0, 2.3 and 0.9% at 0.65, 4.3, 48, 118 and 832 ng/dl, respectively. Inter-assay CVs are 8.9, 6.9, 4.0, 3.6 and 3.5% at 0.69, 4.3, 45, 117 and 841 ng/dl, respectively. The detection range is 0.5–2,000 ng/dl. E2 was measured by LC/MS (Mayo Clinic Laboratories, Mayo Clinic, Rochester, MN) with assay sensitivity of 0.23 to 405 pg/ml, intra-assay CV of 1.4 to 11.8%, and inter-assay CV of 4.8 to 10.8% (6). A1c was assessed by high performance liquid chromatography (Tosoh G8, South San Francisco, CA, USA). Fasting glucose was measured using a Unicel *DxC 800* Auto-analyzer (Beckman Coulter, Fullerton, CA, USA). The following were measured using enzyme-linked immunosorbent assay kits: C-terminal telopeptide of type I collagen (CTx), marker of bone resorption (Crosslaps; Immunodiagnostic System Inc., Gaithersburg, MD), osteocalcin, marker of bone formation, (Metra OC; Quidel Corporation, San Diego, CA), and sclerostin (TECO medical Sclerostin HS Enzyme Immunoassay Kit, Quidel Corp, San Diego, CA). The coefficients of variation (CVs) for the above assays in our laboratory are <10% and <3.5% for A1c (6).

### Statistical Analysis

Results are expressed as means ± SD. A p-value of 0.05 was considered statistically significant. A threshold model was fit to data using non-linear regression with a threshold as an adjustable parameter, optimal threshold A1c = 7%. Patients were grouped into 4 categories based of A1c levels (group I: <6%, group II: 6.0–6.4%, group III: 6.5–6.9%, and group IV: ≥7%). Group comparisons between those with T2D and those without T2D were analyzed using analysis of variance (ANOVA) without and with adjustments for covariates such as age, testosterone levels, estradiol levels, duration of T2D, and medications. Correlations among the different variables were analyzed by simple correlation analysis. The data were managed using Excel 2010 (Microsoft, Redmond, WA) and were analyzed using SAS version 9.3 (SAS Institute, Inc., Cary, NC, USA).

## Results

**Table 1** shows the clinical characteristics of the 217 male participants. The mean age of the participants was 55 ± 9 years old with mean BMI of 36.15 ± 6.44 kg/m^2^, and mean A1c was 6.1 ± 1.5%. Among the 94 subjects with T2D, 15 were not on any medication, 23 were on metformin only, 10 were on insulin only, and 2 were on sulfonylurea only. A total of 29 subjects were on insulin in combination with other agents, the most common combination agent being Metformin (n = 30), followed by sulfonylurea (n = 9). Our study included 2 patients on sodium-glucose co-transporter 2 (SGLT2) inhibitors (Empagliflozin), 7 on Glucagon-like peptide-1 receptor agonsits (GLP1 agonists), 4 on Dipeptidyl peptidase IV (DDP4) inhibitors, 0 on Thiazolidinediones (TZDs), and 15 were on combination with different agents except for insulin.

**Table 1:** Clinical characteristics of the study population according to hemoglobin A1c levels.

Hemoglobin A1c (%)	Group I	Group II	Group III	Group IV	P-value
	<6.0 (n = 90)	6.0–6.4 (n = 48)	6.5–6.9 (n = 24)	≥7.0 (n = 55)	
Age (years)	*52.9 ± 9.5	54.6 ± 8.3	58.5 ± 8.7	58.0 ± 8.7	**0.003**
BMI (kg/m^2^)	35.3 ± 6.4	37.6 ± 7.2	36.4 ± 4.7	36.2 ± 6.0	0.27
Whole Body BMD (g/cm^2^)	1.152 ± 0.110	1.150 ± 0.132	1.182 ± 0.085	1.145 ± 0.110	0.41
Lumbar spine BMD (g/cm^2^)	1.103 ± 0.148	1.132 ± 0.149	1.146 ± 0.127	1.139 ± 0.172	0.85
Total Hip BMD (g/cm^2^)	1.088 ± 0.143	1.111 ± 0.127	1.108 ± 0.093	1.117 ± 0.135	0.95
Femoral Neck BMD (g/cm^2^)	0.888 ± 0.157	0.932 ± 0.153	0.864 ± 0.106	0.882 ± 0.150	0.19
Osteocalcin (ng/ml)	6.92 ± 4.30	6.10 ± 3.18	6.26 ± 2.89	**3.90 ± 2.64	**0.002**
CTx (ng/ml)	0.33 ± 0.17	0.34 ± 0.19	0.30 ± 0.14	**0.18 ± 0.12	**0.0002**
Sclerostin (ng/ml)	0.73 ± 0.27	0.73 ± 0.21	0.77 ± 0.24	0.75 ± 0.21	0.87
Testosterone (ng/dl)	301.74 ± 119.18	295.50 ± 113.74	273.14 ± 92.75	275.73 ± 81.64	0.44
Estradiol (pg/ml)	23.79 ± 24.70	25.58 ± 15.20	20.38 ± 18.32	22.20 ± 14.60	0.79
25-hydroxyvitamin D (ng/ml	26.07 ± 9.31	24.76 ± 10.41	24.33 ± 7.84	25.85 ± 11.59	0.82
PTH (pg/ml)	56.84 ± 28.76	54.01 ± 24.07	55.66 ± 22.47	47.41 ± 29.74	0.30

BMI, body mass index; CTx, C-telopeptide; BMD, bone mineral density; PTH, parathyroid hormone; values for BMD adjusted to age, BMI and testosterone levels; post-hoc analysis for significant comparisons: *p group I vs. III and IV, **group IV vs I, II and III. Bolded p-values are significant.

### Bone Turnover Markers

#### Entire Study Population

**Figure 1:**
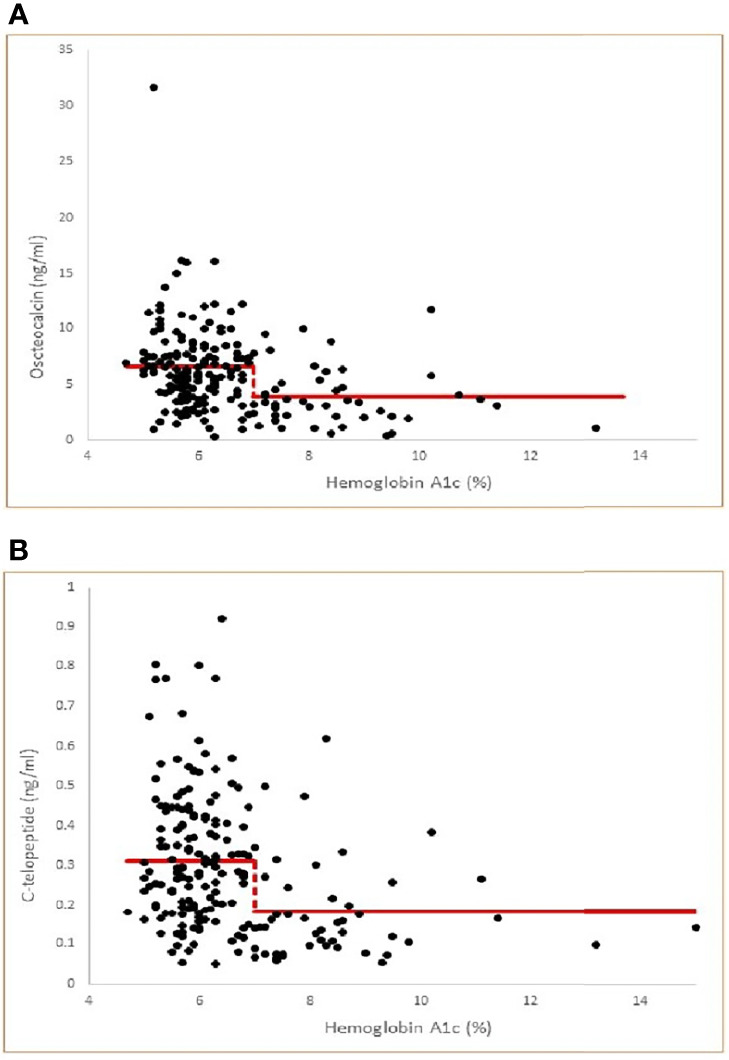
**(A)** Threshold model of Osteocalcin versus Hemoglobin A1c by nonlinear regression (multiple correlation r=0.31, p<0.001) with least squares fitted (optimal) threshold A1c=7.0. Model Osteocalcin values= 6.57 for A1c< 7.0 and 3.86 for A1c ≥ 7.0. The threshold model is statistically significant compared to a null hypothesis of no threshold (P<0.001). **(B)** Threshold model of C-telopeptide versus Hemoglobin A1c by nonlinear regression (multiple correlation r=0.31, p<0.001 with least squares fitted (optimal) threshold A1c=7.0. Model C-telopeptide values= 0.311 for A1c < 7.0 and 0.182 for A1c ≥ 7.0. The threshold model is statistically significant compared to a null hypothesis of no threshold (p<0.001).

We hypothesized that there is an A1c cut-off above which bone turnover markers will show significant reduction. To determine A1c cut-offs for lower osteocalcin and CTx levels, we used a threshold model and nonlinear regression. This shows that an A1c cut-off of 7.0, among all choices of A1c cut-offs, yields the least sum of squared errors in each case (See **Figures 1A, B**).

**Figure 2:**
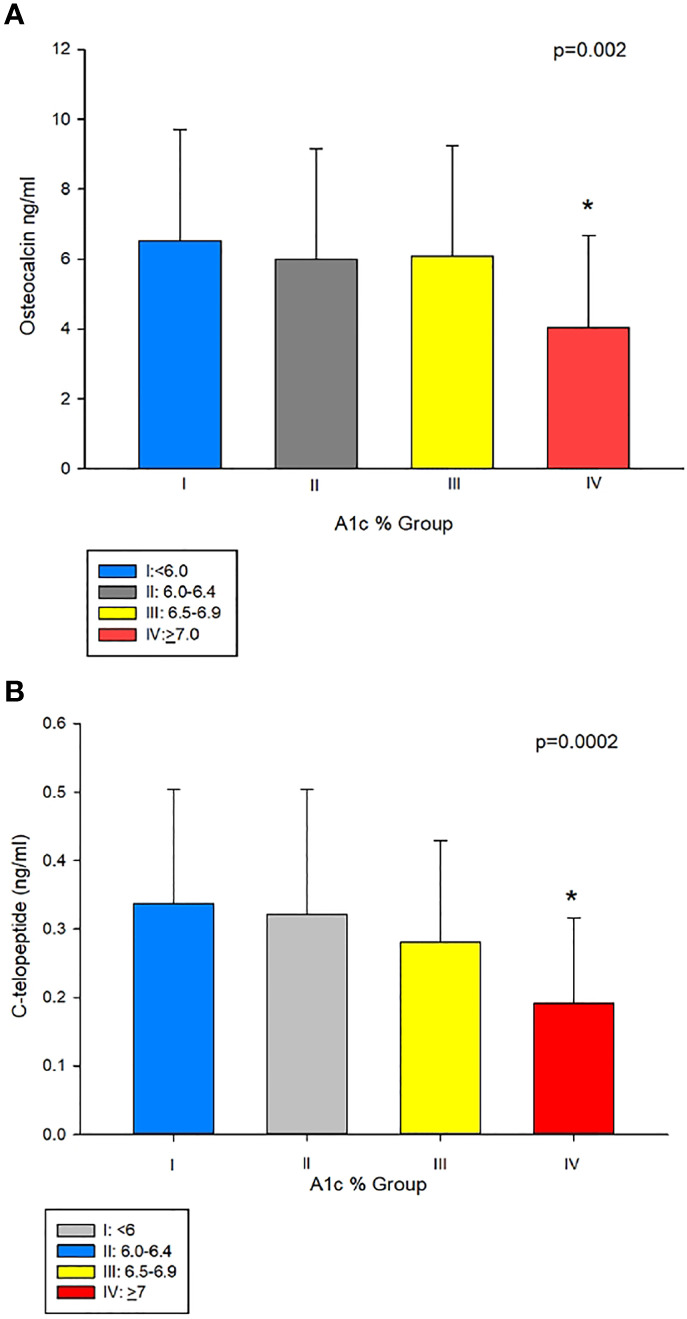
**(A)** Osteocalcin (OC) in group IV (A1c<7%), compared to groups with A1cs ≥7%, i.e. I II, and III (4.04 ± 2.64 vs 6.53 ± 3.18, 5.99 ± 3.16 and 6.09 ± 3.16 ng/mL, respectively, p = 0.002). **(B)** C-telopeptide (CTx) was lower in group IV compared to groups I, II, and III (0.1 9 ± 0.12 ng/mL vs 0.34 ± 0.17, 0.32 ± 0.18 and 0.28 ± 0.14 ng/mL, respectively, p=0.0002). *Post-hoc analysis: p<0.05 showed significant difference in OC and CTx in group IV compared group I, II and III.

To further examine this hypothesis, we divided our subjects into 4 groups based of A1c values (%) (group I: <6, group II: 6.0–6.4, group III: 6.5–6.9, and group IV: ≥7) modified according to clinical guidelines for good control which is <7 as per American Diabetes Association (14) and <6.5 as per American Association of Clinical Endocrinology (15). **Table 1** shows the clinical characteristics of the study population according to A1c grouping. Those in group I were younger than in groups II, III, and IV, the difference of which was significant compared to groups III and IV. As shown in **Table 1** and **Figure 2A**, patients in group IV (with A1c of ≥7%) had significantly lower osteocalcin levels compared to groups I, II, and III (with A1c of <7%) (p = 0.002). CTx was also significantly lower in group IV compared to groups I, II, and III (p = 0.0002) (**Table 1** and **Figure 2B**). Posthoc analysis showed no significant difference in osteocalcin and CTx levels between groups I, II, and III. There were no significant differences in sclerostin levels across the different A1c groups.

#### Subjects With T2D

**Figure 3:**
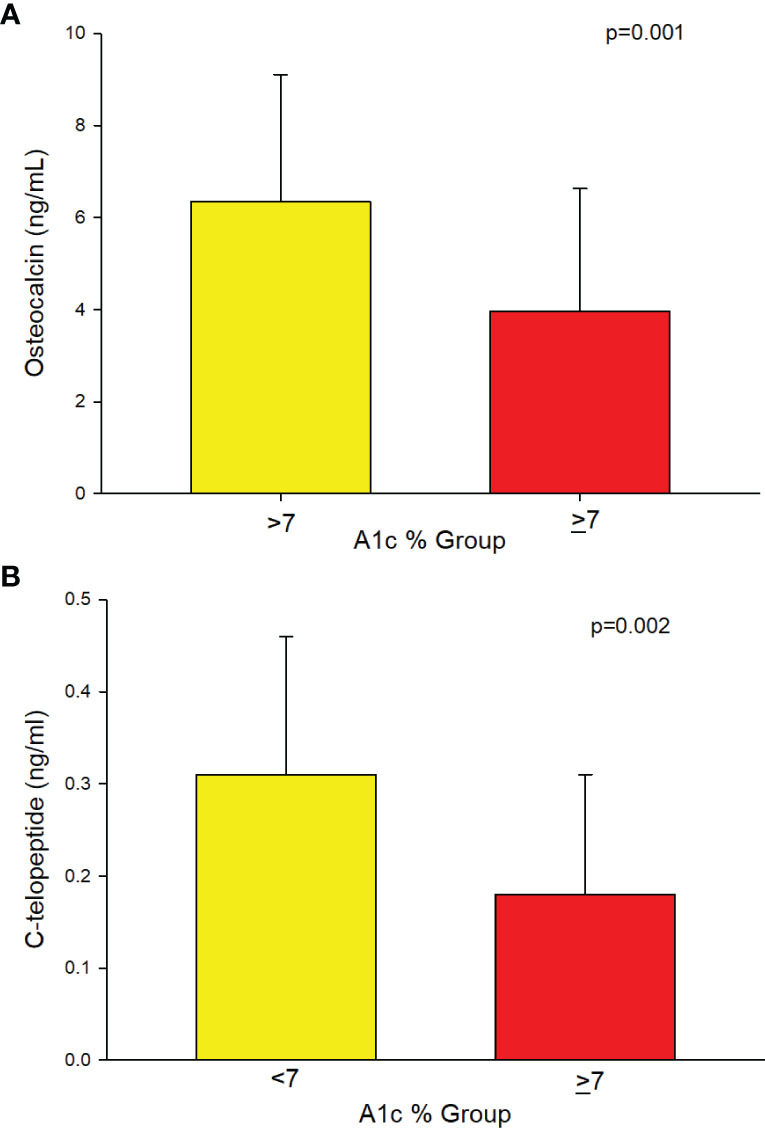
**(A)** Analysis of the subgroup of men with T2D (n=94) showing lower Osteocalcin (3.95 ± 2.68 vs. 6.34 ± 2.77, p=0.001) and **(B)** showing lower C-telopeptide (0.18 ± 0.13 vs. 0.31 ± 0.15, p=0.002) in those with A1c ≥7% compared to those ≤7%, respectively.

A separate analysis of a subset of patients with T2D (n = 94) showed that those with A1c of ≥7% had significantly longer duration of T2D compared to those with A1c of <7% (**Table 2**). Significantly lower osteocalcin (p=0.001), (**Table 2**, **Figure 3A**) and CTx (p <0.002), (**Table 2** and **Figure 3B**) were also observed in those with A1c ≥7% compared to those <7%. The significance between the groups persisted even after adjusting for duration of T2D (p = 0.02 for osteocalcin and p = 0.01 for CTx) and for medication use, (p = 0.02 for osteocalcin and p = 0.05 for CTx), see **Supplemental Table 1**. Analyses adjusted for total testosterone and estradiol showed significantly lower osteocalcin and CTx in subjects with A1c ≥7% than those with A1c of <7% (p <0.001 for both osteocalcin and CTx), see **Supplemental Table 1**. Mean PTH level did not differ between the patients with T2D who have A1c of <7% compared to those with A1c of ≥7% with or without adjustment for 25-hydroxyvitamin D level. There was no significant difference in sclerostin levels between those with A1c of <7% and those with A1c of ≥7% (**Table 2**).

**Table 2:** Clinical characteristics of the participants with T2D according to hemoglobin A1c levels.

Hemoglobin A1c (%)	<7%(n = 39)	≥7%(n = 55)	P
Age	59.1 ± 7.1	58.0 ± 8.7	0.53
BMI	36.2 ± 4.9	36.2 ± 6.0	0.94
Duration of T2D (years)	4.1 ± 3.8	8.7 ± 5.7	**0.0002**
Use of medication for T2D	25/38	53/55	**0.0001**
Whole Body BMD (g/cm^2^)	1.171 ± 0.104	1.145 ± 0.110	0.11
Lumbar spine BMD (g/cm^2^)	1.155 ± 0.149	1.125 ± 0.167	0.50
Total hip BMD (g/cm^2^)	1.108 ± 0.106	1.117 ± 0.135	0.80
Femoral neck BMD (g/cm^2^)	0.871 ± 0.127	0.882 ± 0.150	0.58
osteocalcin (ng/ml)	5.90 ± 2.66	3.90 ± 2.64	**0.001**
CTx (ng/ml)	0.28 ± 0.16	0.18 ± 0.12	**0.002**
Sclerostin (ng/ml)	0.80 ± 0.24	0.75 ± 0.21	0.34
Testosterone (ng/dl)	276.7 ± 117.7	275.7 ± 81.6	0.96
Estradiol (pg/ml)	22.0 ± 18.2	22.2 ± 14.6	0.97
25-hydroxyvitamin D (ng/ml)	25.0 ± 8.9	25.9 ± 11.6	0.72
PTH (pg/ml)	53.8 ± 20.0	47.4 ± 29.7	0.30

BMI, body mass index; T2D, type 2 diabetes mellitus, CTx, C-telopeptide; SCL, Sclerostin; BMD, bone mineral density; PTH, parathyroid hormone; p-values for BMD are adjusted for age, BMI and testosterone levels. Bolded p-values are significant.

Simple correlation analysis between A1c and the different clinical and laboratory parameters in patients with T2D showed negative correlations for both osteocalcin (r = −0.29, p = 0.008) and CTx (r = −0.22, p = 0.048), (**Table 3**). There were also significant negative correlations between osteocalcin and duration of T2D (r = −0.25, p = 0.04) and the use of medications for T2D (r = −0.32, p = 0.003). Similarly, negative correlations were observed between CTx with the duration of T2D (r = −0.35, p = 0.003) and the use of medications (r = −0.41, p = 0.0001). There was no correlation between sclerostin and A1c; but it is significantly positively correlated with age (r = 0.42, p = 0.002) and 25-hydroxyvitamin D (r = 0.26, p = 0.03), see **Table 3**.

**Table 3:** Simple correlation analysis between osteocalcin and CTX with the different Clinical and laboratory parameters in patients with T2D.

	Osteocalcin		CTx		Sclerostin	
	r	p	r	p	r	p
Age (years)	0.14	0.21	0.16	0.14	0.42	**0.002**
BMI (kg/m^2^)	−0.02	0.85	−0.02	0.90	0.14	0.22
Duration of T2D (years)	−0.25	**0.04**	−0.35	**0.003**	0.17	0.16
Hemoglobin A1c (%)	−0.29	**0.008**	−0.22	**0.048**	−0.07	0.57
Testosterone (ng/dl)	0.09	0.41	−0.02	0.87	−0.11	0.37
Estradiol (pg/ml)	−0.07	0.59	−0.06	0.66	−0.21	0.12
25-hydroxyvitamin D (ng/ml)	0.06	0.59	0.06	0.69	0.26	**0.03**
PTH (pg/ml)	0.21	0.09	0.21	0.09	0.03	0.80
Use of medications for T2D	−0.32	**0.003**	−0.41	**0.0001**	0.21	0.07

BMI, body mass index; T2D, type 2 diabetes mellitus, CTx, C-telopeptide; BMD, bone mineral density; PTH, parathyroid hormone. Bolded p-values are significant.

To evaluate the effect of the use of medications for T2D on the different bone biomarkers, we divided our patients with T2D into those who are on medications and those who are not (**Table 4**). As expected, those on medications had T2D for significantly longer period of time and had significantly higher A1c than those not on medications (**Table 4**). More importantly, those on medications had significantly lower osteocalcin and CTx levels compared to those not on medications (**Table 4**). To further evaluate the effect of medications on bone turnover of subjects that are at goal with their T2D, we analyze the subgroup of subjects who have A1c of less than 7% (n = 38) of which 15 were not on medications and 23 were on different antidiabetic medications. There was no significant difference in osteocalcin levels between those who were vs. those who were not on medications (5.55 ± 3.0 vs 6.53 ± 1.91 ng/ml, p = 0.31); and no significant difference in CTx levels between those who were vs. those who were not on medications (0.26 ± 0.16 vs 0.32 ± 0.16 ng/ml, p = 0.29).

**Table 4:** Clinical and laboratory parameters according to the use of medications for T2DM.

	Without medicationsN = 15	With medications N = 79	P
Age (years)	56.0 ± 9.9	58.9 ± 7.7	0.21
Duration of T2D (years)	2.02 ± 3.2	7.7 ± 5.4	**0.003**
Hemoglobin A1c (%)	6.60 ± 0.68	7.95 ± 1.74	**0.004**
Osteocalcin (ng/ml)	6.64 ± 2.0	4.40 ± 2.90	**0.01**
CTx (ng/ml)	0.39 ± 0.15	0.21 ± 0.13	**0.0001**
Sclerostin (ng/ml)	0.66 ± 0.18	0.79 ± 0.23	0.07
PTH (pg/ml)	61.77 ± 27.72	47.77 ± 26.02	0.12

T2D, type 2 diabetes mellitus; CTx, C-terminal telopeptide of collagen I; PTH, parathyroid hormone. Bolded p-values are significant.

### Bone Mineral Density (BMD)

BMD analysis on the spine, total hip and femoral neck adjusted for age, BMI, and T levels showed no significant difference in areal BMD at all sites in patients based on A1c categories (**Table 1**) and in the subset of men with T2D (**Table 2**).

## Discussion

Our results show that in men with T2D, an A1c ≥7% is associated with low bone turnover suggesting that impairment in bone metabolism in these patients starts around the cut-off established by the American Diabetes Association as goal for good control (14). The cut-off of <7% for A1c is established as a goal to minimize or reduce the risk for microvascular (retinopathy, nephropathy, and neuropathy) and possibly also macrovascular complications (MI or coronary artery disease and stroke) common among patients with poorly-controlled T2D. Similar to the other organs in the body, our results suggest that the potential harmful effects of poor glycemic control on bone among patients with T2D likely also starts around this A1c level.

Bone disease associated with T2D is characterized by skeletal fragility despite a relatively normal or higher than normal BMD (2, 3, 16). Although a few studies reported increased cortical porosity by high-resolution peripheral quantitative computer tomography, some reported preserved trabecular bone microarchitecture (17). Thus, the increased fracture risk in T2D is likely from a defect in bone quality rather than bone quantity. There are several mechanisms proposed as contributors to skeletal fragility in patients with T2D and include but not limited to: accumulation of advanced glycation end products (AGEs) which can adversely affect biomechanical properties (18), reactive oxygen species(ROS), use of antidiabetic medications, and the presence of microvascular complications (2, 4); there could be more of these factors present in those with poorly-controlled T2D. However, low bone turnover has become the hallmark of bone derangement associated with T2D with reduced bone formation as the primary defect (5). This abnormality is confirmed from studies in both human and animal models (5, 7, 19, 20). Two large meta-analysis demonstrated significantly lower CTx and osteocalcin in people with T2D compared to those without T2D (7, 21). Transiliac crest bone biopsies in 5 patients with T2D and 4 subjects without T2D revealed reduction in osteoid surface, bone formation rate, osteoblast surface, mineralizing surface, and cortical width T2D compared to those withoutT2D (5). Moreover, circulating osteoblast precursors were also lower in patients with T2D compared to those without T2D. There were also significant reduction in serum markers of bone formation such as osteocalcin and procollagen I intact N-terminal (P1NP) coupled with decreased serum marker of bone resorption, i.e., CTx, in patients with T2D compared to those without T2D (5). These findings suggest inactive bone remodeling which results in failure to repair microcracks and replace old with new bone leading to increase buildup of bone with poor quality.

Our study shows negative correlation between blood glucose control (as assessed by A1c) and bone turnover markers, i.e., higher A1c is associated with reduction in bone turnover markers. Furthermore, our study also identifies an A1c of 7% as the threshold by which reduction in bone turnover occurs among those with T2D. A prior report suggested that T2D patients with low bone formation marker (N-amino terminal propeptide of type 1 procollagen or P1NP) have higher risk for osteoporotic fractures (20). Given the importance of bone remodeling in bone health maintenance, it is critical to identify and cut-off for blood sugar control at which reduction in markers of bone turnover occurs. Although the test accuracy for A1c could be affected by conditions that affect red blood cell turnover, non-enzymatic glycation of hemoglobin, assay variablity and ethinicity, for the most part, it is still considered as a reliable index of the average blood glucose over a period of 12 weeks and has been used in most studies to investigate the effect of glycemic control on target organs. Sclerostin positively correlated with both age and 25-hydroxyvitamin D; associations which have been previously reported (22, 23). On the other hand, there was no significant difference in sclerostin levels across the A1c groups in the entire study population and in those with T2D between A1c of <7% and those with A1c of ≥7%.

Several studies have examined the relationship between blood glucose control using A1c and fracture risk (24–31). While some reported linear relationship between fractures and A1c, others did not (27, 31, 32). Others also showed that there was a significant interaction between the use of insulin and A1c such that, those who are on insulin with tight glycemic control had a higher risk for any clinical fracture (29, 30). These findings were hypothesized due to higher incidence of hypoglycemic events resulting in falls.

A secondary analysis of the Action to Control Cardiovascular Risk in Diabetes (ACCORD) trial failed to find a difference in the incidence of nonvertebral fractures over a period of 3.8 years of follow-up between those in the intensive glycemia and standard glycemia groups (32). While most of the above studies showing an increased risk for fracture with poor glycemic control are done in the elderly population (mostly in their 70s), the ACCORD trial had relatively younger participants, i.e., in their 60s which may partially contribute to the variable findings. In addition, the median A1c, was 6.4% among those in the intensive treatment group, while it was 7.5% in the standard group which suggests that the standard treatment group is not really in poor glycemic control. Although one can say that these inconsistent findings can also be due to a host of factors such differences in assessing glycemic control (single vs. multiple A1c measurements), and the cut-offs used, duration of T2D, diabetes complications, BMI, and fall risks, there seems to be more evidence suggesting that glucose control could play a role in preventing adverse bone complications.

Our results also demonstrated that in the entire population, those T2D patients with A1c of 7% or better (group III and some in group II) have bone turnover (osteocalcin and CTx) that is not different from those without T2D, i.e., group I. While this finding suggests that not all patients with T2D will have low bone turnover, this also implies that it is not the diagnosis of T2D itself but the glycemic control that determines bone complications from T2D. This is supported by the findings that those with T2D with A1c of <7% have significantly higher bone markers than those with ≥7%. Although it appears that medications may have an effect on bone turnover markers, an analysis in those with A1c of <7% showed that there was no significant difference in bone markers between those who were, and were not on medications, suggesting that subjects who were on anti-diabetic drugs have low bone turnover because of poor glucose control rather than from the treatment. In addition, whereas some of our subjects are hypogonadal, and low testosterone (with consequent low estradiol level) may alter bone turnover (33), our analyses are adjusted for testosterone and estradiol levels. Of note, there is no significant difference in testosterone and estradiol levels between those with A1c <7% compared to those with ≥7%.

It is likely that the suppression in bone turnover, the hallmark of diabetic bone disease, occurs much earlier than the structural and biomechanical changes that predispose them to fractures. A1c is a more stable marker of glycemic control compared to fasting and postprandial blood glucose levels, hence, finding an A1c threshold by which bone turnover become suppressed will be useful in identifying who could be potentially predisposed to future skeletal complications from T2D. To our knowledge, there are no other studies to date in men with T2D looking at threshold A1c at which bone health gets affected. Some studies have demonstrated differences in fracture risk between men and women with T2D (34–36). It is possible that there are gender differences in what constitutes as the threshold A1c for bone complications. However, in these studies no particular A1c threshold was reported.

Our study has limitations. We used a single timepoint A1c, i.e., only at the time of baseline visit, thus, we have no data on the long-term blood sugar control of our subjects. Also, our population is composed only of men. Nevertheless, our study is the first to recognize that the A1c recommended by the American Diabetes Association as target to prevent a host of non-skeletal complications from diabetes (14) is also the same A1c level where bone turnover appears to be reduced in men with T2D compared with lower A1c levels in a cross-sectional setting where A1c is measured in a single time point.

## Data Availability Statement

The original contributions presented in the study are included in the article/**Supplementary Material**. Further inquiries can be directed to the corresponding author.

## Ethics Statement

The studies involving human participants were reviewed and approved by the Institutional Review Boards of the University of New Mexico and of the Baylor College of Medicine. The patients/participants provided their written informed consent to participate in this study.

## Author Contributions

SJ and RA-V: conceptualization. SJ, CQ, and RA-V: formal analysis. SJ, DV, and RA-V: investigation. SJ, EB, FD, GG, AF-G, GC, LA, RC, VR, DV, and RA-V: writing, reviewing and editing. All authors contributed to the article and approved the submitted version.

## Funding

This study was supported by the VA Merit Review 101CX000424 and NIH R01 HD093047. Clinical Trial Information: Clinical trial registration number NCT: 01378299 and NCT: 03490513.

## Author Disclaimer

The contents of this manuscript do not represent the views of the U.S. Department of Veterans Affairs or the United States Government.

## Conflict of Interest

The authors declare that the research was conducted in the absence of any commercial or financial relationships that could be construed as a potential conflict of interest.

## Publisher’s Note

All claims expressed in this article are solely those of the authors and do not necessarily represent those of their affiliated organizations, or those of the publisher, the editors and the reviewers. Any product that may be evaluated in this article, or claim that may be made by its manufacturer, is not guaranteed or endorsed by the publisher.
