# Supplementary material for: Hemoglobin A1c Threshold for Reduction in Bone Turnover in Men With Type 2 Diabetes Mellitus
Source: Front Endocrinol (Lausanne). 2021 Dec 28;12:788107. doi: 10.3389/fendo.2021.788107 (PMC8750620; doi:10.3389/fendo.2021.788107)
Supplement: Supplementary file 1 [file Table_1.docx]

| **Supplemental Table 1**. Bone Turnover markers according to A1C in men with T2D adjusted for the different covariates. | | | |
| --- | --- | --- | --- |
| Adjustments of variables | A1c <7% | A1c ≥7% | P value |
| **Duration of Diabetes** |  |  |  |
| Osteocalcin (ng/ml) | 5.58± 0.55 | 3.92± 0.40 | **0.02** |
| CTx (ng/ml) | 0.27±0.026 | 0.18±0.019 | **0.001** |
| **Medication use** |  |  |  |
| Osteocalcin (ng/ml) | 5.60±0.46 | 4.07±0.38 | **0.02** |
| CTx (ng/ml) | 0.26±0.02 | 0.19±0.02 | **0.05** |
| **Total Testosterone and estradiol** |  |  |  |
| Osteocalcin (ng/ml) | 6.52±0.33 | 3.86±0.58 | **<0.001** |
| CTx (ng/ml) | 0.32±0.02 | 0.17±0.03 | **<0.001** |
| Values are means ± SE, CTx: C-terminal telopeptide of type 1 collagen | | | |
